# Liquid Biopsy for Colorectal Adenoma: Is the Exosomal miRNA Derived From Organoid a Potential Diagnostic Biomarker?

**DOI:** 10.14309/ctg.0000000000000356

**Published:** 2021-05-12

**Authors:** Tomoyuki Handa, Masatake Kuroha, Hiroshi Nagai, Yusuke Shimoyama, Takeo Naito, Rintaro Moroi, Yoshitake Kanazawa, Hisashi Shiga, Yoichi Kakuta, Yoshitaka Kinouchi, Atsushi Masamune

**Affiliations:** 1 Division of Gastroenterology, Tohoku University Graduate School of Medicine, Sendai, Japan;; 2 Department of Gastroenterology, Shirakawa Kosei General Hospital, Fukushima, Japan;; 3 Health Administration Center, Center for the Advancement of Higher Education, Tohoku University, Sendai, Japan.

## Abstract

MicroRNAs (miRNAs) can serve as tumor biomarkers; however, their role in evaluating colorectal adenoma (CRA) is unclear. Recently, the organoid culture system enabled long-term expansion of human colon epithelium. This study aimed to examine the potential of exosomal miRNAs extracted from CRA organoids as biomarkers in the clinical liquid biopsy CRA test.

**METHODS:**

We established organoid cultures from normal colon and CRA using resected specimens. Exosomes were isolated from the conditioned medium organoids. MiRNAs were isolated from the exosomes, and their expression profiles were compared using microarray analysis. To identify miRNA candidates for liquid biopsy, we prospectively compared changes in their expression in serum and exosomes before and after endoscopic resection in 26 patients with CRA.

**RESULTS:**

Seven exosomal miRNAs were overexpressed in CRA organoids: miR-4323, miR-4284, miR-1268a, miR-1290, miR-6766-3p, miR-21-5p, and miR-1246. The expression levels of 4 exosomal miRNAs (miR-4323, miR-4284, miR-1290, and miR-1246) and 2 serum miRNAs (miR-1290 and miR-1246) were significantly lower in posttreatment sera. The combined expression of 4 exosomal miRNAs could identify both CRA and large-size (>12.6 cm^2^) CRA with respective areas under the curve of 0.698 (95% confidence interval [CI] = 0.536–0.823) and 0.834 (95% CI = 0.660–0.929). Combinations of 2-serum miRNA expression values could identify both CRA and large-size CRA with respective area under the curves of 0.691 (95% CI = 0.528–0.817) and 0.834 (95% CI = 0.628–0.938).

**DISCUSSION:**

We found that exosomal miRNAs derived from the CRA organoid culture could be potential diagnostic biomarkers for CRA.

## INTRODUCTION

Colorectal cancer (CRC) is the third most common malignancy and the second leading cause of cancer-related death worldwide (1). CRC follows a linear progression from a normal colonic epithelium to adenoma initiation, carcinoma transformation, and metastasis (2). Therefore, the detection and treatment of colorectal adenomas (CRA) can significantly reduce the risk of invasive colorectal tumors and mortality (3). For early CRC detection, stool-based screening with a fecal immunochemical test is commonly used worldwide (4). However, these tests have low sensitivity for adenomatous lesions (7.6%–40%) (5–7) and are unsuitable for CRA screening. In addition, colonoscopy is unsuitable as a screening test either because of high invasiveness, cost, and low adherence (8). A noninvasive and highly sensitive screening CRA test is urgently needed.

Recent studies have demonstrated that microRNAs (miRNAs) are secreted from various cells, including cancer cells, into bodily fluids such as blood, urine, breast milk, and saliva, either as free miRNAs or in exosomes (9). miRNAs are noncoding single-stranded RNA molecules, between 18 and 23 nucleotides in length, which promote mRNA cleavage and subsequent degradation by binding to the complementary 3′ untranslated region of the mRNA (10). It was shown that miRNAs have been implicated in the development and progression of CRC by functioning as oncogenes and tumor suppressors (11). Exosomes are 50–100 nm vesicles that contain diverse host cell-derived bioactive molecules such as proteins, lipids, and miRNAs (12). There has been increasing interest in exosome-miRNAs as candidates for liquid biopsy biomarkers. Reportedly, serum exosome miRNAs such as let-7a, miR-1229, miR-1246, miR-150, miR-21, miR-223, miR-23a, and miR-486 were highly expressed in patients with CRC and regarded as potential biomarkers for use in liquid biopsy (9). Nevertheless, few reports addressed the use of miRNA in liquid biopsy for CRA screening. One reason is that candidate miRNAs for liquid biopsy have been isolated from the culture supernatant of colon cancer cell lines originating from advanced colon cancer.

Development of 3-dimensional organoid culture systems has supported long-term expansion of human normal colon crypts, CRA, and CRC (13,14). In organoid cultures, cancer stem cells are maintained in a microenvironment using a cocktail of niche factors supplemented in the medium, including Noggin, R-spondin, and epidermal growth factor. For this study, we aimed to investigate profiles of exosome miRNAs derived from CRA using a long-term culture in 3-dimensional organoids. Furthermore, we examined whether candidate miRNAs derived from CRA-derived organoids (CRA organoids) have the potential to be used as diagnostic biomarkers for CRA.

## MATERIALS AND METHODS

### Patients

This study examined patients who had been diagnosed with CRA of more than 10 mm in diameter. Furthermore, all lesions were treated using endoscopic submucosal dissection. This study included data of 54 patients at Tohoku University Hospital between February 2018 and April 2019. Colorectal tumors were defined as adenoma in categories 3 and 4 and as cancer in category 5 according to the Vienna Classification (15). Patients were excluded from this study if they met 1 or more of the following criteria: history of cancer in other organs, inflammatory disease such as connective tissue disease, and diagnosed as category 5 by histological assessment after endoscopic submucosal dissection. This study was approved by the Ethics Committee of Tohoku University Hospital (nos. 2017-1-949 and 2019-1-379). All patients provided written informed consent before participation.

### Research method

**Figure 1.**
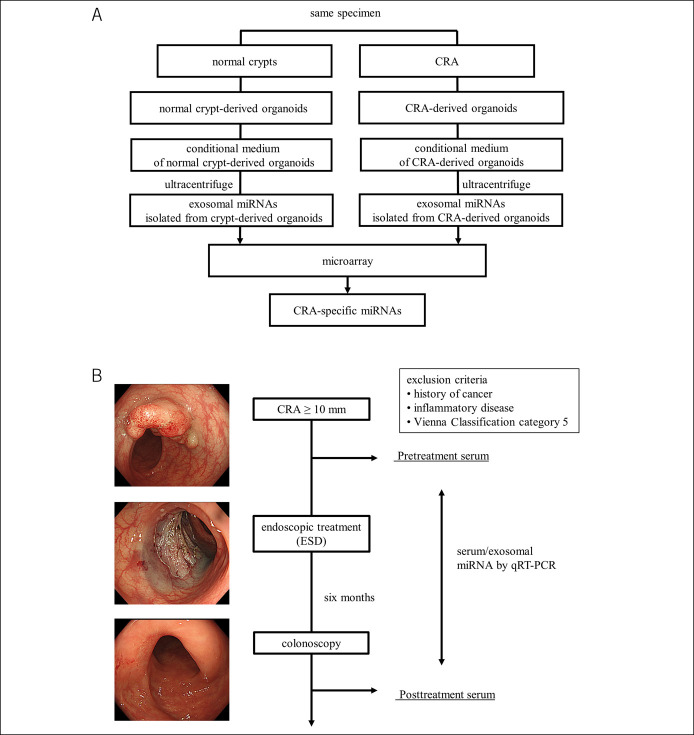
Study design overview. (**a**) Selection of candidate miRNAs using the organoid culture. Organoids from the normal colon crypts and CRA are established in pairs from endoscopically resected specimens. Then, the exosomes are isolated from conditional medium collected from each organoid using ultracentrifugation. Then, microarray analysis is conducted for each of the exosomal miRNAs isolated from the normal colon crypt-derived organoids and CRA-derived organoids. CRA-specific miRNA candidates are extracted. (**b**) Flow diagram showing the strategy for qRT-PCR analysis of serum miRNAs before and after endoscopic resection. Pretreatment sera are obtained from patients with CRA. Then, CRA is resected using ESD within 1 month. Posttreatment sera are obtained 6 months after resection. At that time, total colonoscopy has been performed to confirm healing of the mucosa and lack of recurrence or residual tumor in the treated area. The expression changes between pretreatment sera and posttreatment sera of candidate miRNAs are validated using RT-PCR. CRA, colorectal adenoma; miRNAs, microRNAs; ESD, endoscopic submucosal dissection; qRT-PCR, quantitative reverse transcription-polymerase chain reaction.

This study was conducted in 2 phases. In the first phase (Figure 1a), we established organoids from the normal colon crypts and CRA in pairs using endoscopically resected specimens from 3 patients. Then, exosomes were isolated from conditional medium collected from each organoid using ultracentrifugation. Then, microarray analysis was conducted for exosomal miRNAs isolated from the normal colon crypt-derived organoids and from CRA-derived organoids. In the second phase (Figure 1b), we prospectively compared changes in the expression of serum and exosomal miRNA after endoscopic resection. Pretreatment sera were obtained within 1 month before endoscopic resection. Posttreatment sera were obtained at 6 months after endoscopic resection. At that time, total colonoscopy was performed to confirm healing of the mucosa and lack of recurrence or residual tumor in the treated area. The candidate miRNAs selected in the first phase were validated using real-time polymerase chain reaction (RT-PCR).

### Establishment of organoid cultures

Organoid culture was established using previously published protocols (16) with slight modifications. In brief, tumor tissues were cut and carefully washed with ice-cold phosphate buffered saline (PBS). The tumor tissues were then incubated in a 37 °C water bath for 30 minutes with 50 μg/mL Liberase (TH research grade; Roche Diagnostics, Basel, Switzerland). Tumor cell pellets were then resuspended in Matrigel (BD Matrigel; Becton, Dickinson, Franklin Lakes, NJ) and seeded onto 24-well plates. For normal colon tissues, the tissues were cut into small pieces and were then washed carefully with ice-cold PBS. Normal tissue fragments were then incubated with 2.5 mM EDTA chelation buffer for 1 hour at 4°C. Next, crypts were released by violently pipetting with PBS. The crypts were then resuspended in Matrigel and were seeded onto 24-well plates. Both the normal colon and tumor organoids were cultured using organoid culture medium with niche factor advanced DMEM/F12 (Gibco, Grand Island, NY) with penicillin–streptomycin (Gibco), 2 mM GlutaMAX (Gibco), 10 mM HEPES (Gibco), 2% B-27 Supplement (Gibco), 1 mM N-acetylcysteine (Wako, Osaka, Japan), 50 ng/mL EGF (Gibco), 100 ng/mL Noggin (Peprotech, Rocky Hill, NJ), 10% R-spondin-1 conditioned medium (Trevigen, Gaithersburg, MD), 10% Afamin/Wnt3a (JSR Life Sciences, Tsukuba, Japan), 500 nm A83-01 (Wako), 10 nm Gastrin (Sigma-Aldrich, St. Louis, MO), and 10 μM SB202190 (Cayman Chemical, Ann Arbor, MI). In addition, 10 μM Y-27632 (Wako) was added to each well to the culture medium for establishment of organoid cultures after passage. The medium was replaced every 2–3 days, and organoids were passaged, generally on every seventh day of culture.

### Exosome isolation from organoid-conditioned medium

Exosomes were isolated from conditioned medium collected from organoids using ultracentrifugation (17). After several passages, the organoid-conditioned medium was collected. The medium was first centrifuged at 300*g* for 10 minutes and then at 2000*g* for 30 minutes to precipitate the cells. The supernatant was then filtered through 0.2 μm filters and ultracentrifuged at 100,000*g* for 70 minutes to pellet exosomes, which were then washed with PBS. These vesicles were then ultracentrifuged at 100,000*g* for 70 minutes. The final pellet was resuspended in 100 μL PBS and frozen at −80 °C.

### Electron microscope analysis

Isolated exosomes were evaluated for their morphology and size distribution using transmission electron microscopy, as previously described (18). Exosomes were fixed with 2% glutaraldehyde in 0.1 M phosphate buffer and were subsequently fixed in 2% osmium tetroxide for 2 hours. After washing with distilled water, dehydration through a graded series of ethanol, and embedding in epoxy resin at 60 °C, ultrathin sections were prepared using an ultramicrotome (LEICA EM UC7; Leica Microsystems Japan, Tokyo, Japan). They were then stained with uranyl acetate and lead citrate and examined using a transmission electron microscope (TEM, Hitachi H-7600; Hitachi High-Technologies, Tokyo, Japan).

### Nanoparticle tracking analysis

The amount and size distribution of exosomes were analyzed using a microscope (Nanosight NS300; Malvern Panalytical, Malvern, United Kingdom). Each sample was diluted appropriately with PBS to give counts in the linear range of the instrument. The particles in the laser beam exhibit Brownian motion; video was recorded in duplicate for 60 seconds. Data were then analyzed using NTA software (ver. 3.3; Malvern Panalytical).

### RNA isolation and microarray analysis

Total RNAs including miRNAs were extracted using a kit (miRNeasy Mini Kit; Qiagen, Hilden, Germany) according to the manufacturer's instructions. The RNA quality was assessed by using a microfluidic chip (Bioanalyzer 2100 Expert; Agilent Technologies, Santa Clara, CA) using a kit (RNA 6000 Pico Kit; Agilent Technologies). For microarray analysis (Agilent SurePrint G3 Human miRNA Microarray Kit 8 × 60 K, ver. Human_miRNA_V21,0; Agilent Technologies), 2 ng total RNAs from the exosome fractions were labeled using kits with a miRNA labeling reagent (Low Input Quick Amp Labeling Kit, RNA spike kit; Agilent Technologies). Then, they were hybridized according to the manufacturer's instructions. After hybridization, the array was washed and scanned using a DNA microarray scanner (Agilent Technologies). After numerical conversion of the raw data using feature extraction software (ver. 10.7; Agilent Technologies), the transformed data were analyzed using software (GeneSpring ver. 12.5; Digital Biology).

### MiRNA and exosomal miRNA extraction from patients' serum

From each participant, whole blood was taken and processed using centrifugation at 1900*g* for 10 minutes. To remove cell debris, the supernatant was centrifuged at 4 °C and 16,000*g* for 10 minutes and then stored at −80 °C. Total miRNA was extracted from serum samples as described above. Exosomes were extracted from serum samples by using the polymer precipitation method (ExoQuick; System Biosciences, Palo Alto, CA). Then, exosomal RNA was extracted as described above.

### Quantitative RT-PCR analysis of miRNAs

Serum concentrations of 6 candidate miRNAs were quantified by reverse transcription polymerase chain reaction (RT-PCR) using a kit (Taqman MicroRNA Reverse Transcription; Applied Biosystems, Foster City, CA) and Taqman miRNA Assay (Applied Biosystems). The RT-PCR was performed in triplicates using a LightCycler 96 system (Roche, Basel, Switzerland). The miR-16 was used as an internal control (19), and miRNA expression levels were normalized by miR-16 using the 2−ΔCt method.

### Statistical analyses

Statistical analyses were performed using JMP ver. 14.0 (SAS Institute, Tokyo, Japan). To compare miRNA expression in paired samples, *t* tests were used. The values are presented as mean ± SD. Significance was inferred for results found to have 2-sided *P* < 0.05. In graphs showing the relative expression of each miRNAs in the patient sera, diagnostic performance for each biomarker individually and in combination was evaluated using receiver operating characteristic (ROC) curve analysis and by reporting the area under the curve (AUC).

## RESULTS

### Isolation of exosomal miRNA from patient-derived organoids

**Figure 2.**
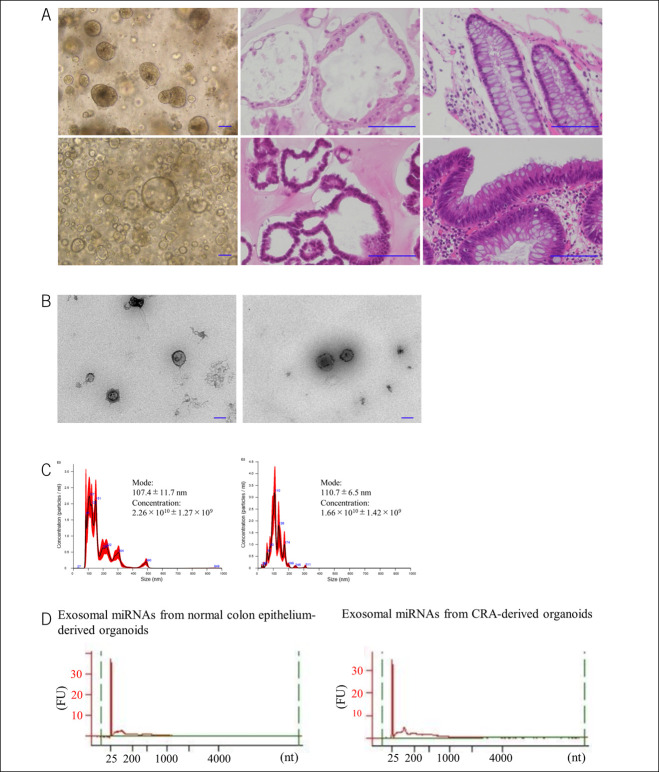
Patient-derived organoids and isolation of exosomes. (**a**) Top panels show the images of normal colon crypts. Optical microscope image of organoids (left), H&E staining of organoids (center), and H&E staining of endoscopically resected specimens (right). Bottom panels show the images of the CRA. Optical microscope image of organoids (left), H&E staining of organoids (center), and H&E staining of endoscopically resected specimens (right). Each scale bar is 100 μm. (**b**) Electron microscope imaging of the isolated exosomes. Images of exosomes from normal colon crypt-derived organoids (left) and of exosomes from CRA-derived organoids (right). Original magnification: ×15,000. Scale bar shows 100 nm. (**c**) Size distribution of isolated exosomes analyzed using a Nanosight NS300. Size distribution of normal colon crypt-derived organoids (left). Size distribution of exosome from CRA-derived organoids (right). (**d**) Exosomal miRNAs are prepared from normal crypt-derived and CRA-derived organoids. Their integrity and quality are examined using a bioanalyzer system. Exosomal miRNA did not include ribosomal RNAs. Image of exosomal miRNAs from normal colon crypt-derived organoids (left), image of exosomal miRNAs from CRA-derived organoids (right). CRA, colorectal adenoma; H&E, hematoxylin–eosin; miRNA, microRNA.

We established 3 organoid lines from normal colon crypts and CRA in pairs using the specimen resected by endoscopy. Paraffin sections of the organoids showed that the established organoids imitated the original structures as a budding form, as elucidated using hematoxylin–eosin staining (Figure 2a). Exosomes were isolated from conditioned medium of the organoids using ultracentrifugation. Under transmission electron microscopy, the size of the vesicles was approximately 100 nm (Figure 2b), which corresponded to the results of earlier studies (12). The nanoparticle tracking analyzer showed that the mode size of the vesicles in isolated exosomes was 107.4 ± 11.7 nm (Figure 2c). Next, we isolated exosomal miRNAs derived from normal colon crypts organoids and CRA organoids. We confirmed that the isolated exosomal miRNAs did not include ribosomal RNA using a Bioanalyzer (Figure 2d).

### Microarray analysis revealed profiles of exosomal miRNA derived from CRA organoids

Microarray analysis was conducted for each of the exosomal miRNAs isolated from the normal colon crypts organoids and CRA organoids. Among exosomal miRNA isolated from CRA-derived organoids, the expression levels of 15 miRNAs were higher, and those of 97 miRNAs were lower than levels of miRNA isolated from normal crypt-derived organoids. In CRA-derived organoids, levels of 10 miRNA (miR-4323, miR-4284, miR-1268a, miR-1290, miR-6766-3p, miR-21-5p, miR-1246, miR-2278, miR-3148, mir-595) were higher, with FC > 0.5; and those of 12 miRNAs were lower, with FC > 3: miR-633, miR-6087, miR-4443, miR-5703, miR-6833-5p, miR-711, miR-7150, miR-6133, miR-4534, miR-6865-5p, miR-6775-5p, and miR-4716-3p (Table 1). From 10 miRNAs showing increased levels in the CRA-derived organoid, we selected 7 candidate miRNAs for the second phase study: 5 miRNAs (miR-4323, miR-4284, miR-1268a, miR-1290, and miR-6766-3p) that were found to have higher levels with FC > 1 and 2 miRNAs (miR-21-5p and miR-1246) that were shown to have association with colon cancer in previous studies (9,20).

**Table 1.** miRNAs for which expression was changed in exosomes between adenoma and normal epithelium by microarray analysis

Exosomal miRNA					
Adenoma/normal			Normal/adenoma		
Systematic name	log_2_ FC	*P* value	Systematic name	log_2_ FC	*P* value
hsa-miR-4323	2.21	0.18	hsa-miR-630	4.71	<0.01
hsa-miR-4284	2.21	0.18	hsa-miR-6087	4.61	<0.01
hsa-miR-1268a	2.06	0.25	hsa-miR-4442	4.23	0.01
hsa-miR-1290	1.41	0.03	hsa-miR-5703	4.11	0.08
hsa-miR-6766-3p	1.29	0.53	hsa-miR-6833-5p	3.47	0.07
hsa-miR-21-5p	0.89	0.70	hsa-miR-711	3.42	<0.01
hsa-miR-1246	0.86	0.09	hsa-miR-7150	3.37	0.08
hsa-miR-2278	0.74	0.63	hsa-miR-6133	3.24	0.08
hsa-miR-3148	0.70	0.75	hsa-miR-4534	3.15	0.18
hsa-miR-595	0.52	0.76	hsa-miR-6865-5p	3.08	0.09
			hsa-miR-6775-5p	3.07	0.10
			hsa-miR-4716-3p	3.06	0.10

FC, fold change; miRNA, microRNA.

### Levels of 4 exosomal miRNAs and 2 serum miRNAs reduce after endoscopic resection

**Figure 3.**
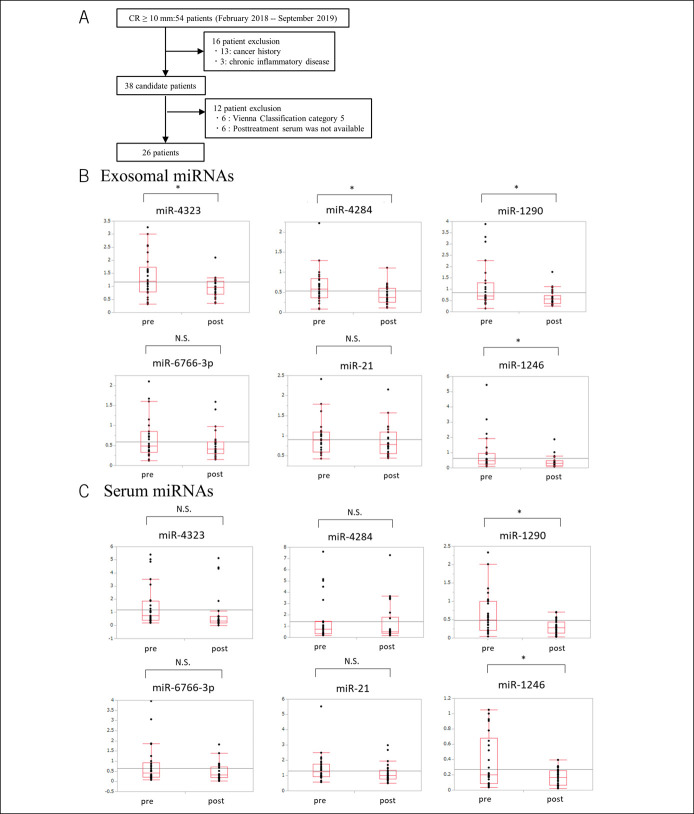
Expression levels of serum and exosomal miRNA before and after endoscopic resection. (**a**) Flow chart of the prospective study shows the differences in miRNA expression before and after endoscopic resection. (**b**) Exosomal miRNA expression levels before and after endoscopic resection in 26 patients. The respective expression levels of miR-4323, miR-4284, miR-1290, and miR-1246 are significantly lower after endoscopic resection. (**c**) Serum miRNA expression levels before and after endoscopic resection in 26 patients. The expression levels of miR-1290 and miR-1246 are significantly lower after endoscopic resection. CRA, colorectal adenoma; pre, before endoscopic resection; post, after endoscopic resection; miRNA, microRNA; N.S., not significant. *P < 0.05.

To clarify the diagnostic value for CRA, we examined the expression of candidate miRNAs in patients with CRA. Initially, 54 patients were enrolled in this study. Then, 13 patients were excluded because they had a history of cancer or inflammatory disease. Another 6 patients were excluded because they were diagnosed as having a tumor of category 5 by histopathological assessment of resected specimens. Six patients were excluded because serum after treatment was not available. Finally, 26 patients were analyzed in this study (Figure 3a). Clinical characteristics of 26 patients are presented in Table 2. Because primers of mir-1268a could not be created with sufficient accuracy (data not shown), we validated the expression changes between pretreatment sera and posttreatment sera of 6 miRNAs using RT-PCR: miR-4323, miR-4284, miR-1290, miR-6766-3p, miR-21, and miR-1246. Among exosomal miRNAs, 4 miRNAs had lower levels in posttreatment serum than in pretreatment serum: miR-1290, miR-1246, miR-4323, and miR-4284 (Figure 3b). Among serum miRNAs, 2 miRNAs (miR-1290 and miR-1246) had lower levels in posttreatment serum than in pretreatment serum (Figure 3c).

**Table 2.** Clinical characteristics of the patients from whom serum was collected before and after endoscopic treatment

Characteristics	Patients (n = 26)
Age, n (%)	
<70 yr	13 (50.0)
≥70 yr	13 (50.0)
Sex, n (%)	
Male	10 (38.5)
Female	16 (61.5)
Location, n (%)	
Right colon	19 (73.1)
Left colon	7 (26.9)
Macroscopic type, n (%)	
Protruding	3 (11.5)
LST-G	15 (57.7)
LST-NG	8 (30.8)
Tumor size, n (%)^a^	
<12.6 cm^2^	13 (50.0)
≥12.6 cm^2^	13 (50.0)
Vienna Classification, n (%)	
Category 3	14 (53.8)
Category 4	12 (46.2)

LST-G, laterally spreading tumor granular type; LST-NG, laterally spreading tumor nongranular type.

a Tumor size was assessed by the elliptical area and was calculated from long and short axes (cm) in resected specimens.

Next, we examined whether high expression of miRNAs was associated with clinical characteristics. In univariate and multivariate analyses, high-expression level of serum mir1290 was associated with adenoma size. No other miRNA (either exosomal or serosal) was found to be associated with clinical characteristics (Table 3).

**Table 3.** Clinical factors with high expression of serum miR-1290

	High expression of serum miR-1290 (n = 13)	Univariate^a^ *P* value	Multivariate^b^	
			OR (95% CI)	*P* value
Age, n (%)				
<70 yr	6/13 (46.1)	1.00		
≥70 yr	7/13 (53.8)			
Sex, n (%)				
Male	5/10 (50.0)	1.00		
Female	8/16 (50.0)			
Location, n (%)				
Right colon	7/19 (36.8)	0.07	Reference	
Left colon	6/7 (85.7)		5.59 (0.26–118.23)	0.27
Macroscopic type, n (%)				
Protruding	1/3 (33.3)	0.14	Reference	
LST-G	10/15 (66.7)		15.6 (0.34–710.67)	0.16
LST-NG	2/8 (25.0)		11.3 (0.15–829.61)	0.27
Tumor size, n (%)				
<12.6 cm^2^	11/13 (84.6)	<0.01	Reference	
≥12.6 cm^2^	2/13 (15.4)		33.1 (1.97–556.64)	0.02
Vienna Classification, n (%)				
Category 3	6/14 (42.9)	0.70		
Category 4	7/12 (58.3)			

Factors with *P* value of less than 0.2 in univariate analysis were included in multivariate analyses.

CI, confidence interval; LST-G, laterally spreading tumor granular type; LST-NG, laterally spreading tumor nongranular type; OR, odds ratio.

a Chi-square test or Fisher's exact probability test.

b Multiple logistic regression analysis.

### Diagnostic ability of candidate miRNAs to serve as biomarkers for CRA

**Figure 4.**
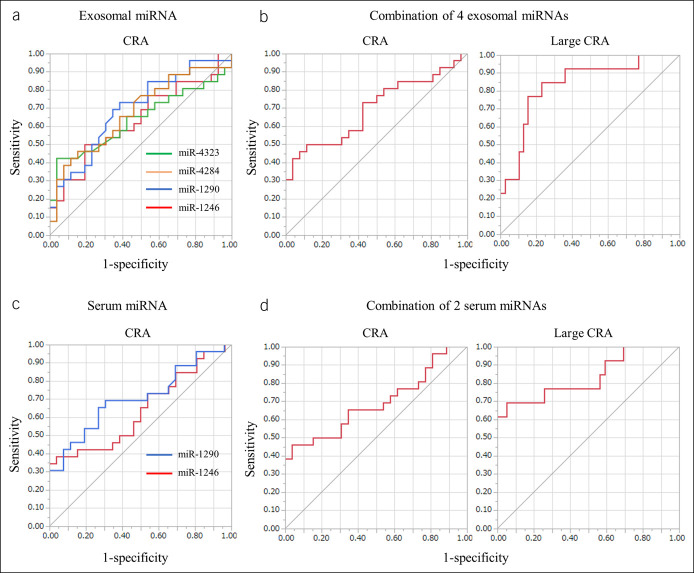
ROC curve analysis using serum or exosomal miRNA levels to distinguish between patients with CRA. (**a**) Exosomal miR-4323, miR-4284, miR-1290, and miR-1246 levels yield respective AUC values of 0.637, 0.677, 0.694, and 0.667 in distinguishing between patients with CRA. (**b**) Combined usage of 4 serum exosomal miRNAs gives AUC values of 0.698 in distinguishing CRA (left) and 0.834 in distinguishing large CRA (approximately ≥12.6 cm^2^) (right). (**c**) Serum miR-1290 and miR-1246 levels yield AUC values of 0.705 and 0.639, respectively. (**d**) Combined usage of 2 serum miRNAs results in an AUC value of 0.691 for CRA (left) and 0.834 for large CRA (right). AUC, area under the curve; blue line, miR-1290; CRA, colorectal adenoma; green line, miR-4323; miRNA, microRNA; red line, miR-1246; ROC, receiver operating characteristic; yellow line, miR-4284.

We generated ROC curves to assess the potential of selected miRNAs to serve as biomarkers for CRA (Figure 4). Regarding exosomal miRNAs, ROC analyses revealed that miR-4323, miR-4284, miR1290, and miR-1246 levels were significantly discriminating factors identifying CRA in patients with respective AUC values of 0.637 (95% confidence interval [CI] = 0.470–0.776), 0.677 (95% CI = 0.514–0.805), 0.694 (95% CI = 0.534–0.818) and 0.635 (95% CI = 0.472–0.772). The AUC for combined values of 4 exosomal miRNAs was 0.698 (95% CI = 0.536–0.823). Next, we attempted to identify patients with large adenoma (approximately ≥12.6 cm^2^; median tumor size), in whom ROC analyses revealed combined values of 4 exosomal miRNAs with AUC values as 0.834 (95% CI = 0.660–0.929).

Regarding serum miRNA, ROC analyses used miR-1290 and miR-1246 levels as significant factors for discriminating the presence of CRA with respective AUC values of 0.705 (95% CI = 0.544–0.827) and 0.639 (95% CI = 0.476–0.776). The AUC for combined values of 2 miRNAs was 0.691 (95% CI = 0.528–0.817). In patients with large adenomas, ROC analyses revealed combined values of 2 serum miRNAs with AUC values of 0.834 (95% CI = 0.628–0.938).

## DISCUSSION

In this study, we extracted exosomal miRNA from the 3-dimensional organoid culture and elucidated profiles of exosomal miRNAs isolated from CRA organoids. Reportedly, the expression levels of exosomal miRNAs such as miR-21, miR-1246, miR-1290, miR-23a, miR-200c, miR-203a, miR-17, miR-19a, and miR-7641 are increased in CRC (21–26). Although expression levels of miR-21, miR-1246, and miR-1290 are reportedly increased in CRC and CRA, no study has described the increased expression of miR-4323, miR-4284, miR-1268a, or miR-6766-3p in CRA. These miRNAs might be related to mutation of the *APC* gene, which causes adenoma initiation in the colonic epithelium (27). The results also suggest that the expression of exosomal miRNAs is altered during progression of colorectal tumors through various stages.

To our knowledge, no other study has examined exosomal miRNAs extracted from the organoid culture as a candidate for liquid biopsy. The AUCs in patients with CRA were 0.698 for the combination of exosomal miRNAs and 0.691 for the combination of serum miRNAs. Moreover, the AUCs in patients with larger CRA (approximately ≥12.6 cm^2^) were 0.834 for the combination of exosomal miRNAs and 0.834 for the combination of serum miRNAs. The AUC in fecal immunochemical test for advanced adenoma (those ≥10 mm size or with villous and/or high-grade dysplasia) is reportedly about 0.62 (6). The diagnostic capacity of liquid biopsy using miRNAs presented in this report is presumed to be higher. Furthermore, the results, demonstrating that better diagnostic capacity was obtained for larger tumors, suggest that miRNAs used as candidates are secreted by tumors. Because it has been reported that the larger was the size of adenoma, the more often it was complicated with cancer (28), it is expected that the candidate miRNAs will be especially useful as a screening tool for adenomas with high risk of cancer. However, the accuracy of liquid biopsy must be improved, particularly in consideration of the labor and costs of miRNA extraction and PCR testing. In this study, exosomes were extracted from the organoid culture supernatant using ultracentrifugation because of the low number of exosomes in the culture supernatant, which required a large amount of medium. On the other hand, in a prospective study using patient sera, the polymer precipitation method was used. The available exosome types differ depending on the separation method (29,30). Presumably, miRNAs can be more effective biomarkers for CRA if they could be isolated exclusively from the tumor-derived exosomes. Indeed, results of our study showed no difference in serum miRNA levels for miR-4283 or miR-4323; however, differences in expression for these 2 miRNAs were observed when they were isolated from exosomes. One influential factor might be that the difference in expression levels was detected with higher accuracy because exosomes were extracted selectively.

Several screening tests for CRC have been developed (31). We considered how liquid biopsy established in this study could contribute to the current clinical framework for CRC screening. The potential to detect CRA which are precancerous lesions of CRC may allow for a more detailed risk stratification to identify high-risk groups for CRC that are indicated for colonoscopy. In addition, because the compliance rate for stool-based tests is generally low, blood-based tests are considered to improve the consultation rate. Furthermore, it has been reported that the profiles of exosomal miRNAs isolated from organoids were different between CRC and CRA (32). If patients with CRC and CRA are found to have different miRNA profiles in their sera, more accurate patient stratification may be possible. The US Multi-Society Task Force recommendations define a follow-up period for surveillance endoscopy for metachronous cancers after colonoscopy and polypectomy according to patient risk (33). However, there is limited evidence for the decision on a particular interval, and further study of alternative testing is required. One modeling study suggested that fecal immunological testing may be effective for surveillance (34). Liquid biopsy is expected to be an alternative to endoscopy screening and help establish an appropriate follow-up period. Nevertheless, further large-scale studies, including those investigating its usefulness and cost-effectiveness, are needed.

In this prospective study, the expression of miRNAs in sera or serum exosomes of patients with CRA was compared before and after endoscopic resection. Although differences in serum miRNAs before and after surgical resection in advanced CRC have been reported (9,35), no report of the literature has compared serum miRNA levels before and after endoscopic resection of adenoma. Because factors other than the tumor can be excluded, such a method can be regarded as yielding more accurate study results. The expression of serum exosomal miR-21, miR-29a, and miR-92a has been suggested for use as biomarkers for patients with CRA (36). In that study, however, no difference was observed for miR-21 in the serum before and after treatment. Moreover, secretion of miR-29a or miR-92a from adenomatous organoids was not observed, possibly because of different extraction methods. In addition, miR-29a and miR-92a might be secreted by cells other than tumor cells, such as fibroblasts and immune cells.

Reportedly, miR-4323 has been expressed less in patients with rectal cancer than in those with normal colorectal epithelium (37), and it has also been expressed less in sera of patients with CRC than in healthy persons (38). One report described that miR-4284 was enriched in the cyst fluid from pancreatic intraductal papillary mucinous neoplasm with low grade dysplasia, but it was depleted in the cyst fluids derived from invasive pancreatic carcinomas (39). The expression of miR-4323 and miR-4284 might be lower in highly malignant tumor. Some reports have described that miR-1290 was expressed at a higher level in patients with CRC (40) and that it was related to epithelial mesenchymal transition (41), with miR-1246 promoting the development of CRC through the expression of TGF-β (42) making it a diagnostically useful biomarker of CRC and recurrence (9,20,43,44).

This study has several limitations. First, this investigation was conducted in a small number of patients, and it was a single center study. Second, studies on tumor lesions other than colorectal tumors are also need to verify that candidate miRNAs are specific for patients with CRA. Finally, although serum at 6 months after endoscopic treatment was used as the control, the time it takes for the serum miRNA levels to return to normal is yet unclear.

In summary, this study elucidated a profile of exosomal miRNAs obtained from the CRA organoid culture. Results suggest that the extracted candidate miRNAs might be applicable as biomarkers in liquid biopsy. Organoid culture has been applied to not only the colorectal tumors but also tumors of various organs (45–47). Using this technology, exosomal miRNAs could be used to identify additional candidate miRNAs to be used in liquid biopsy to screen for tumors in various organs and at different stages.

## CONFLICTS OF INTEREST

**Guarantor of the article:** Masatake Kuroha, MD, PhD.

**Specific author contributions**: T.H. and M.K.: designed the study and wrote the initial draft of the article. Y.K. and A.M.: contributed to the analysis, interpretation of data, and assisted in the preparation of the article. H.N., Y.S., T.N., R.M., Y.K., H.S., Y.K.: contributed to data collection and interpretation and critically reviewed the article. All authors approved the final version of the article and are accountable for all aspects of the work.

**Financial support**: This study was supported by JSPS KAKENHI Grant number JP19K17421.

**Potential competing interests**: None reported.

**Study Highlights**

**WHAT IS KNOWN**

✓ MicroRNAs (miRNAs) have attracted considerable attention as tumor biomarkers.
✓ Few reports on CRA for liquid biopsy are available.
✓ The development of three-dimensional organoids has supported the long-term expansion of human normal colon epithelia.

**WHAT IS NEW HERE**

✓ We elucidated the profiles of exosomal miRNAs from CRA organoids.
✓ Candidate miRNAs derived from CRA organoids have potential significance as diagnostic biomarkers for CRA.

**TRANSLATIONAL IMPACT**

✓ Organoid culture systems can be applied to tumors of various organs.
✓ Exosomal miRNAs could be isolated from different tumors to serve as candidates for liquid biopsy.
